# Guatemala scientific and professional diaspora in Spain: an initial characterization

**DOI:** 10.3389/frma.2026.1860284

**Published:** 2026-07-16

**Authors:** Kleinsy Bonilla, Claudia S. Romero-Oliva, Susana Arrechea, Juan Manuel Castillo-Zamora, Marie André Destarac

**Affiliations:** 1 Observatorio Económico Sostenible (OES), Universidad del Valle de Guatemala, Guatemala City, Guatemala; 2 Organization for Women in the Developing World, Trieste, Italy; 3 New Sun Road Guatemala, Guatemala City, Guatemala; 4 University of Valladolid, Valladolid, Spain; 5 Royal Academy of Engineering, Madrid, Spain

**Keywords:** brain linkage, Guatemala, international cooperation, migration, science advice, scientific diasporas, Spain, student mobility

## Abstract

Scientific and professional diasporas can strengthen science, technology, and innovation systems in their home countries, especially in emerging economies with limited infrastructure. These diasporas facilitate knowledge circulation, transnational collaboration, and science diplomacy. In Guatemala, interest is growing in its highly qualified diaspora, especially when host countries like Spain share historical and linguistic ties. This study offers an initial profile of the Guatemalan scientific and professional diaspora (GSPD) in Spain, using a mixed-methods approach: database creation, an online questionnaire, and a focus group. The sample consists mostly of Guatemalan professionals with postgraduate degrees, in early or mid-career, concentrated in major cities such as Barcelona, Madrid, and Valencia. Student mobility—driven by scholarships and aid, funded by both Spanish and local, and international sources—emerges as a key migration factor. Participants report a strong commitment to maintaining ties with Guatemala, collaborating with peers, and sharing expertise through mentorship and institutional engagement. However, they face hurdles such as distrust, lack of recognition, and limited formal mechanisms for collaboration from Guatemalan institutions. These barriers stem from insufficient institutional mandates, poor coordination, and low visibility of the GSPD as a collective actor. The study also notes structural inequalities in international mobility, with mestizo/ladino participants overrepresented and Indigenous populations underrepresented among highly skilled migrants. Rather than viewing migration only as “brain drain,” the findings emphasize brain linkages and diaspora knowledge networks. Strengthening institutional mechanisms—including science diplomacy and collaboration platforms—could foster more inclusive and sustainable scientific development in Guatemala. As an exploratory study with a non-probabilistic sample, these findings provide an initial analytical basis for future research.

## 1 Introduction

Guatemalans pursue postgraduate education to overcome economic limitations and improve their job prospects. However, Guatemala's higher education and scientific ecosystem present structural challenges. Limited research funding, inadequate laboratory infrastructure, uneven graduate program quality, and weak institutional linkages combined with broader socioeconomic constraints, compels many Guatemalans to pursue higher education abroad (Bonilla and Kwak, 2014; UNESCO, 2017; Bonilla and Kwak, 2015; Bonilla et al., 2025). A census conducted by the Guatemalan National Secretariat of Science and Technology (SENACYT) shows up to 68% of Guatemalan doctoral students and graduates studied abroad (SENACYT, 2017). After graduation, a third of postgraduate students choose to remain abroad due to the lack of academic or industry job opportunities in Guatemala (Bonilla and Bin, 2018), becoming part of the Guatemala scientific and professional diaspora (GSPD).

For decades, Guatemala has relied heavily on international cooperation and scholarship programs to develop its scientific and professional workforce. This reflects broader asymmetries in global knowledge production and access to advanced scientific training (Bonilla and Kwak, 2014, 2015; Bonilla and Bin, 2018; Bonilla et al., 2025). Academic international mobility influences the formation of scientific diasporas. Research on scientific diasporas challenges traditional “brain drain” perspectives by emphasizing concepts such as brain circulation, brain linkages, diaspora knowledge networks, transnational collaboration, and science diplomacy (Meyer, 2011; UNESCO, 2006; Echeverría-King L et al., 2022). Within these frameworks, highly qualified members of the diaspora should not represent a loss of human capital for countries of origin, but the configuration of potential actors in transnational processes of knowledge exchange, mentoring, institutional collaboration, and scientific cooperation. The Guatemala science and professional diaspora (GSPD) could play a pivotal role in building Guatemala's scientific and professional ecosystem. Many in the GSPD want to interact with their peer communities in Guatemala and contribute to national knowledge generation. Recent valuable, though limited, efforts have been made to identify, map, and characterize this highly qualified diaspora (Bonilla et al., 2022; Bonilla and Bin, 2018). However, further research is needed to study the GSPD and understand the specifics of the region or the country where they live. The presence of GSPD in Spain is a particularly relevant case study because of the historical, linguistic, academic, and cooperation ties between both countries, as well as Spain's longstanding role as a destination for Guatemalan postgraduates. Examining the GSPD in Spain also reveals how international academic mobility remains shaped by structural inequalities related to class, ethnicity, access to scholarships, and institutional prestige.

Diaspora profiles provide valuable information for decision making, but generating them is hindered “by unclear definitions, low visibility, trust issues, limited institutional capacity, high costs, and fragmented engagement structures” (EUDF European Union Diaspora Facility, 2024; 6). The GSPD's potential remains largely untapped because neither countries of origin nor countries of destination systematically collect data on location, composition, aspirations, assets, and contributions. To better understand the GSPD, this research observed the small-scale diaspora dynamics in Spain which illuminate broader migration patterns. This initial characterization focused on explaining and understanding trajectories and proposing ways forward to reduce gaps between the diaspora and their connections and collaborations with their host country and Guatemala.

The questions guiding this research were: (i) What characteristics are common to the GSPD in Spain?; (ii) What are the mobility trends and dilemmas experienced by the GSPD in Spain?; (iii) What does the GSPD do and where in Spain?; (iv) What conclusions can be drawn from the experiences of the GSPD in Spain and its connection with Guatemala?; (v) Which mechanisms/experiences are diaspora members using to contribute to knowledge production in Guatemala?; and (vi) Which barriers and enabling factors limit or support sustained collaboration and knowledge transfer between Spain and Guatemala?

To answer these questions, a mixed-methods approach was used that included in-depth interviews, archive review, and an online questionnaire followed by a focus group discussion. This enabled a description of migration trajectories, professional contributions from the sample studied, and potential for sustained collaboration with Guatemala. Significant components of the Spain-Guatemala cooperation include providing higher education scholarships for Guatemalans to pursue higher education in universities located in Spain, trends in student mobility, the formation of the GSPD in Spain, and rich qualitative data collected from GSPD members.

## 2 Context

The Guatemala–Spain academic mobility corridor is part of broader transnational dynamics of highly skilled migration, scientific mobility, and knowledge circulation between emerging and high-income economies. These mobility processes are shaped by educational opportunities and international cooperation mechanisms, and by structural inequalities related to class, ethnicity, institutional prestige, and unequal access to global academic networks. Spain has become an important destination for Guatemalan postgraduate mobility and for the emergence of a segment of the GSPD. International cooperation between Spain and Guatemala has undergone a continuous, multifaceted evolution. It was first formalized in 1977 (Spain, Secretaría de Estado de Cooperación Internacional, 2025). The *Spanish Official Development Assistance* (ODA) emphasized restoring democratic institutions in Guatemala following periods of military rule in the 1980s and early 1990s. Signing the *Agreements for a Firm and Lasting Peace* in 1996 was a milestone that ended a long-standing internal armed conflict and provided a concrete framework for action by the international community (Gómez Ramírez, 2012). Later, this cooperation expanded to other sectors, primarily social infrastructure and services. Importantly, Spain has consistently supported educational and training programs for Guatemalans to study in Spanish Universities. Spain classifies Guatemala as a priority partnership country in the *Spanish Cooperation Master Plans* (Cooperación Española., 2026). The most recent planning instrument, the Sixth *Master Plan for Spanish Cooperation* (2024–2027), reaffirms Guatemala as a middle-income partner country. The Spanish Agency for International Development Cooperation's (AECID) sectoral priorities in Guatemala are clearly defined; they seek to address the country's structural challenges: malnutrition, gender violence, weak rule of law, and human rights violations.

Scholarship programs represent some of the most stable and strategic pillars of Spanish cooperation in Guatemala. Managed primarily by the Carolina Foundation (FC) and AECID, this instrument focuses on long-term investment in human capital, creating knowledge networks, and training academic elites committed to development. Educational opportunities offered by the Spanish cooperation are grouped under the framework of the *Spanish Cooperation Scholarships* and integrate the calls for applications from the FC and the AECID. The awardee selection process considers merit and ability and prioritizes equity and social impact by seeking candidates who demonstrate a proactive attitude toward contributing to the common good in their countries of origin. According to FC's official statistics (Fundación Carolina, 2026), the foundation awarded 439 scholarships to Guatemalans between 2001 and 2025 across postgraduate, doctoral, postdoctoral, and mobility initiatives.

Guatemalans' student mobility from 2000 to 2025 comprises three categories with distinct patterns across educational levels and socioeconomic profiles: undergraduate studies, in-person postgraduate studies, and online postgraduate programs with a short in-person component in Spain. These academic programs represent mechanisms of transnational human capital circulation, and each embodies different rationales, selection logics, and expected returns on investment.

- Foundation scholarships (such as FC) operate under public diplomacy and development cooperation frameworks. They prioritize candidates with academic excellence and demonstrate potential to strengthen institutional capacity in their countries of origin.
- Government bilateral programs from the Ministry of Foreign Affairs, European Union, and Cooperation of Spain (MAEC) through AECID follow state-to-state cooperation logic. They balance diplomatic relations with technical needs assessment to target strategic development sectors.
- Corporate social responsibility initiatives such as those from the Santander Universities. They pursue brand positioning alongside regional talent development to create long-term alumni networks that enhance corporate influence.
- Regional university consortia (AUIP) emphasize inter-institutional partnerships. They consolidate academic networks across Ibero-America.
- University-specific programs are talent attraction strategies that align with each institution's internationalization targets and program-specific excellence criteria.
- Doctoral grants aim to build research ecosystems. They select candidates with established publication records to form scientific diasporas capable of knowledge generation.
- Specific programs for the medical and dental fields focus on health sector professionalization. They target clinical excellence to modernize healthcare delivery systems.
- Loan-scholarship models such as *Guatefuturo* (PCB Guatefuturo, 2026) represent human capital investment approaches that tie financial support to repayment capacity and return commitments.
- It is also worth considering the mobility of Guatemalans who pursue higher education studies in Spain with self-funded programs.

In 2023, the Secretariat of Planning and Programming of the Presidency (SEGEPLAN),—the government agency responsible by law for administering the scholarship and academic aid for Guatemalans- recorded a total of 24,162 international scholarships awarded. These were supported by various cooperating sources, including Spain (SEGEPLAN Guatemala, 2023). The current Spanish cooperation strategy is rigorously framed within the 2030 *Agenda for Sustainable Development Goals* (SDGs) and is guided by the global “triple transition”: digital, ecological, and social development (AECID—Agencia Española de Cooperación Internacional para el Desarrollo, 2014). Several institutional actors in Guatemala and Spain could facilitate engagement with the GSPD. However, their interactions with current and future GSPD members remain fragmented, *ad hoc*, and devoid of strategic coordination. These assets remain insufficiently documented and integrated into national strategies. Consequently, Guatemala lacks a coherent framework for effectively utilizing this highly qualified workforce. This limits its potential contribution to scientific, technological, and broader development objectives.

Universities in Guatemala, graduate offices, and alumni programs sporadically connect with their overseas alumni. However, these efforts rarely develop beyond initial outreach. The most effective example is Universidad del Valle de Guatemala (UVG) where alumni programs remain in contact with former students who reside overseas, including in Spain (Universidad del Valle UV and Unidad de Egresados, 2026). At the national level, three institutions have responsibility for characterizing, mapping, and engaging with the GSPD: The Ministry of Foreign Affairs (MINEX), the National Secretariat of Science and Technology (SENACYT), and SEGEPLAN ([Bibr B25][Bibr B5]).

- The Ministry of Foreign Affairs (MINEX) operates through embassies and consulates that provide basic consular assistance to Guatemalan students abroad. It does not have academic attachés or science/technology officers to cultivate professional networks or facilitate bidirectional knowledge flows.
- SENACYT manages science and technology funding, scholarship programs, and innovation priorities. It also disseminates calls for postgraduate training abroad. However, it does not systematically aim for specific cooperation profiles, nor does it track recipients or integrate their expertise into national research agendas post-departure.
- SEGEPLAN coordinates development planning and international cooperation and channels donor funds to advanced training opportunities for Guatemalan students. However, without a cohesive national strategy for diaspora re-engagement, it relies heavily on external partners to define scholarship priorities (Woo and Reyes, 2018).

Collectively, these institutions initiate *ad hoc* support for outbound talent but fail to evolve these relationships into intentional, long-term partnerships with the GSPD.

## 3 Methodology

**Figure 1:**
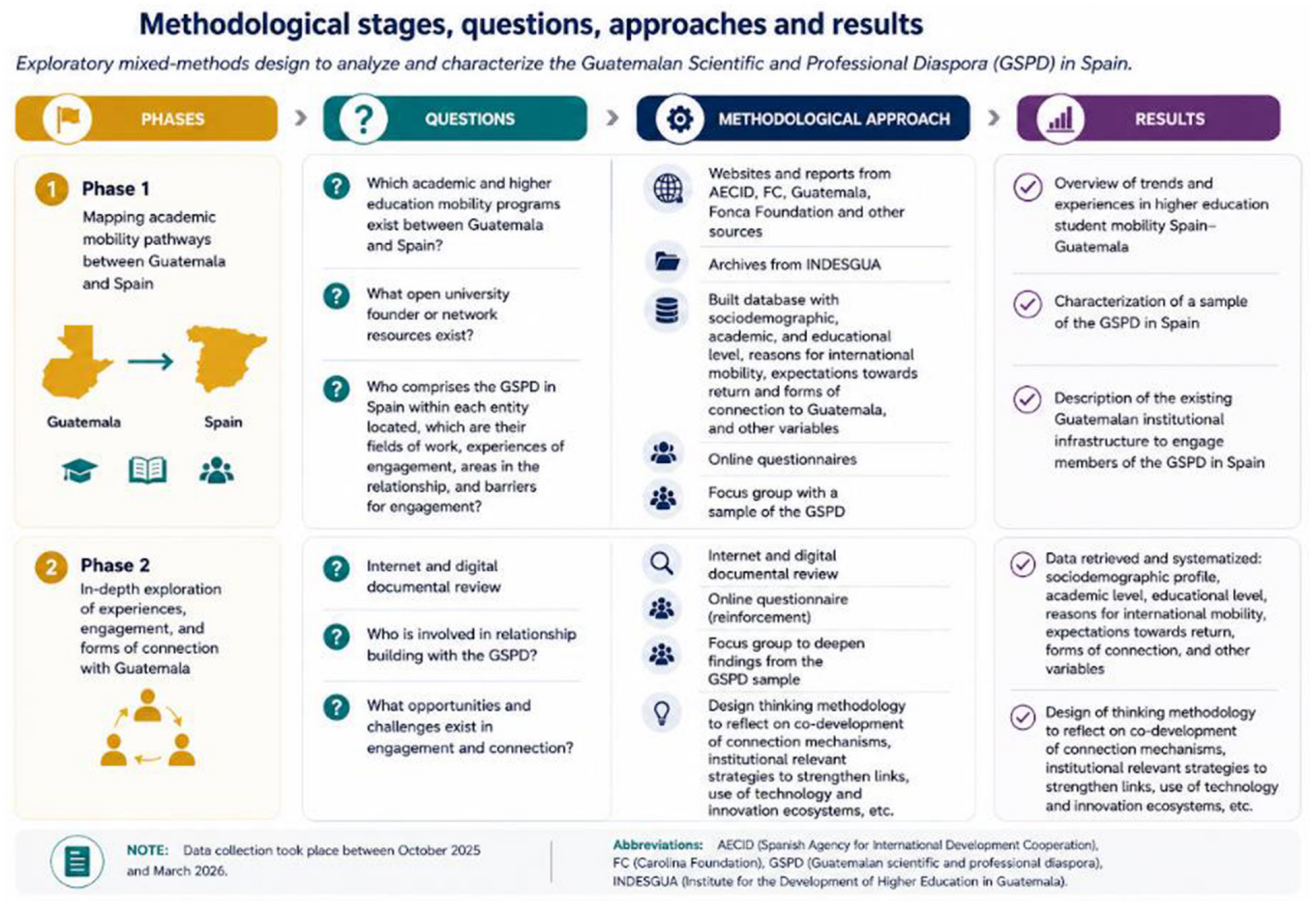
Methodological stages, questions, approaches and results. Exploratory mixed-methods design to analyze and characterize the Guatemalan Scientific and Professional Diaspora (GSPD) in Spain.

This study adopted an exploratory mixed methods design to analyze and characterize the GSPD in Spain. The research combined quantitative and qualitative approaches to identify patterns of bilateral mobility, disciplinary composition, career trajectories, and forms of connection with Guatemala. Data was collected between October 2025 and March 2026. The study was divided into Phase I: obtain and analyze available information and develop a database and mapping of the GSPD in Spain, and Phase II: gather the experiences of members of the GSPD in Spain (Figure 1).

The mapping and database were developed by publishing an open invitation to engage in this study via internal networking and emails. It was open to all on social media channels such as LinkedIn, GoogleScholars, ResearchGate from several accounts including those of the researchers, SENACYT, and the International Network of Science and Technology of Guatemala (RedCTi). The study targeted individuals with the following characteristics: **(i)** Guatemalan national; **(ii)** having resided for over 1 year in Spain prior to the date of the study; **(iii)** engaged in professional/highly qualified activities; and **(iv)** possess knowledge transfer links in Guatemala. A historical background of higher education student mobility was constructed via in-depth interviews and archive review with the Institute for the Development of Higher Education in Guatemala (INDESGUA). To better understand views, motivations, and behavior of the GSPD in Spain, participants were invited to complete an online questionnaire via social media and digital channels. Sections of this online questionnaire included: eligibility (criteria to be considered member of the GSPD), demographic information (gender, age, ethnic identification), characterization and mapping (type of mobility and reasons for migration, geographical preference, and motivations for returning to Guatemala or staying abroad), and linking and engagement with Guatemala (existing mechanisms to connect with Guatemala, known and perceived institutions who interact with the GSPD, challenges and obstacles to connect and engage with Guatemala). Qualitative and quantitative data retrieved from these sources contributed to the identification of demographic characteristics of the GSPD sample, their known and perceived transnational collaboration networks, means for knowledge transfer, and other connections to Guatemala.

Qualitative data was obtained from three primary sources: in depth-interviews (*n* = 2), focus group (*n* = 25), and open-ended questionnaire responses (*n* = 51). These were analyzed through thematic categories to identify recurring narratives related to mobility patterns, institutional barriers in Spain and Guatemala, professional integration in Spain and upon return to Guatemala, and ongoing transnational engagement with Guatemala. Quantitative data were used to determine the demographic profiling (i.e., geographical location, age, gender, socioeconomic status, etc.).

Because participation relied primarily on professional networks, digital dissemination, and voluntary participation, the sample likely overrepresents highly connected, institutionally engaged, and digitally visible members of the GSPD. Consequently, less connected, precarious, rural, or marginalized diaspora members and profiles may be underrepresented. This limitation is particularly relevant in relation to ethnic and socioeconomic inequalities shaping access to international mobility and participation in transnational professional networks.

## 4 Results

The following observations are based on the literature review, in depth interviews (n=3), database analysis (*n* = 111), and online questionnaires (*n* = 51).

### 4.1 Student mobility Guatemala-Spain 2000-2025

Spain-Guatemala student mobility in higher education from 2000 to 2025 can be reviewed in three categories: undergraduate studies, in person postgraduate studies, and online postgraduate programs and online programs with a short in person component in Spain.

#### 4.1.1 Undergraduate studies

Undergraduate studies involve a mix of self-financing, institutional scholarships, and targeted programs. This primarily attracts students from urban middle to upper socioeconomic backgrounds and from prestige alumni networks from two major regions: Guatemala metropolitan region and Quetzaltenango (Arenass Luis Edgar, 2025a). Key drivers include alumni networks, flexible private university admissions, and post-pandemic shifts away from costlier options such as renowned universities in the United States of America. While public universities in Spain have led in scholarships over the past decade, private institutions have increased their influence due to easier calendar alignment for international school graduates.

#### 4.1.2 In-person post graduate studies

Historically, Guatemalan student mobility to Spain has been concentrated at the postgraduate level, with master's programs more common than doctoral degrees. In most cases, doctoral candidates have had prior training abroad to attain research experience and publications. Except for scholarships awarded by the University of Valladolid, the University of Jaén, and the University of Huelva (which have also benefited graduates of the University de San Carlos de Guatemala [USAC]), most master's scholarships are obtained by graduates of Guatemalan private universities (Arenass Luis Edgar, 2025b). This is partly explained by the fact that the USAC evaluation system makes it difficult to achieve high academic averages, while most Spanish universities and programs prioritize academic excellence as a selection criterion.

Scholarship programs and institutions influence the dynamics of postgraduate student mobility. The FC scholarships rank among the most competitive and widely recognized by Guatemalans seeking academic programs in Spain. Complementary cooperation agreements—such as those between USAC and University of Santiago de Compostela (USC) in Agricultural and Environmental Sciences, and Erasmus+ mobility in international law (exchanges, short-term, and full programs)—have further supported this type of student mobility.

MAEC-AECID scholarships include a master's program for government officials, local governments, administrative and teaching staff of public universities, and members of the diplomatic service. However, schemes targeting civil servants are complex for Guatemala, due to unstable work conditions which lack conditions to take leave of absence for academic training. Santander Bank and Santander Universities scholarships support a broad network of public and private Spanish universities, but usually only offer partial financial aid. Guatemalans therefore target institutions with more comprehensive coverage like the Universities of Salamanca, Valladolid, Alicante, Alcalá, Huelva, León, Burgos, Rovira i Virgili, Autonomous University of Madrid, and University of Jaén. Among these, Salamanca, University of Jaén, and Valladolid lead historically by awarding at least one scholarship to a Guatemalan on yearly basis. The Ibero-American University Association for Postgraduate Studies (AUIP) offered master's and doctoral scholarships between 2008 and 2025 to Latin American citizens from member universities. Initially, these were only partial scholarships, but are now full scholarships (excluding airfare) via the Federico García Lorca Program to Andalusian public universities, Valencia, and Cantabria. Universidad Rafael Landívar (URL) and USAC became members of AUIP in 2022 and have since awarded an estimated 12 to 15 scholarships. Several Spanish universities offer scholarships from their own or administered funds to open applicants. Many of these are partial, but some are complete, often with the support of Guatefuturo.

Non-university aid includes Madrid City Council residence scholarships and Ministry of Science and Innovation grants at *Residencia de Estudiante*s. The ministry launched the Industrial Doctoral Program in 2011 (BOE Boletín Oficial del Estado, 2011) to support students researching within companies. Many recipients were subsequently offered jobs at the same or similar firms upon completing their degrees. From 2008 to 2025, Guatefuturo offered a Scholarship-Loan Program that supported 163 Guatemalans studying in Spain (PCB Guatefuturo, 2026). Its repayment requirements mean that recipients are primarily from upper-middle and upper socioeconomic sectors. Most beneficiaries graduate from private universities such as Universidad Francisco Marroquín (UFM), UVG, URL, and Universidad del Istmo (UNIS).

The USAC has developed multiple programs for academic exchange and postgraduate education for researchers and professors in Spain. Information on the fields of knowledge, units of implementation and outcomes of such exchanges is scattered, unsystematized and outdated. (Arenass Luis Edgar, 2025c). Areas of medical and dental sciences are the most common targets for postgraduate studies in Spain— via scholarships, Guatefuturo, or self-financing—due to language compatibility and structured access. Medical specialties require the competitive Medical Residency Exam (MIR) which necessitates costly in-person preparation courses. Successful residents earn stipends and most recipients stay in Spain upon completion. Dental specialties (except Oral/Maxillofacial Surgery via MIR) use university-specific master's admissions after degree validation. Upper-middle and upper socioeconomic sector graduates (mainly from UFM) typically return to Guatemala for lucrative local practice.

The Ford Foundation International Fellowship Program (IFP) provided 4,000 scholarships to support graduate education for social and political leaders globally. In Guatemala, the program was administered by the Center for Regional Research of Mesoamerica (CIRMA) from 2001 to 2010 (CIRMA Centro de Investigaciones regionales de Mesoamérica, 2012). The IFP awarded 127 postgraduate scholarships targeting lower socioeconomic background and minority indigenous applicants. The program was permanently discontinued globally in 2014. Spain was a primary European destination due to language and historical ties, though exact numbers are unavailable.

#### 4.1.3 Online postgraduate courses and online programs with a short in-person stay in Spain

Over the past decade, Spanish universities and business schools have offered postgraduate scholarships (mainly master's) via agreements with the Organization of American States (OAS), SEGEPLAN, and other intermediary organizations. These are either 100% online or include brief in-person components. While many other institutions offer online programs, Guatemalan recipients primarily attend FUNIBER/European University of the Atlantic, Educational Format Business School, International University of La Rioja (UNIR), European School of Management and Business (EUDE), International University of Valencia, European University, and European University Miguel de Cervantes Business School. Again, this type of mobility is mostly concentrated in middle-upper and upper socioeconomic sectors of Guatemala.

### 4.2 Characterization of the GSPD in Spain

Three research instruments (database, questionnaire, and focus group) were used to identify patterns in demographic composition, academic background, disciplinary areas, motivations for migration, and forms of transnational participation of the GSPD in Spain. While the sample is not representative of the entire Guatemalan population in Spain, it offers an initial empirical approximation of the characteristics of a highly qualified segment of this community. It identifies capacities, networks, and potential mechanisms for transnational collaboration (International Organization for Migration, 2019).

#### 4.2.1 Sociodemographic profile and geographic presence in Spain

The geographical distribution of the GSPD in Spain shows a clear concentration in Barcelona, Madrid, and Valencia—Spain's major urban centers and primary economic, academic, and administrative hubs.

**Figure 2:**
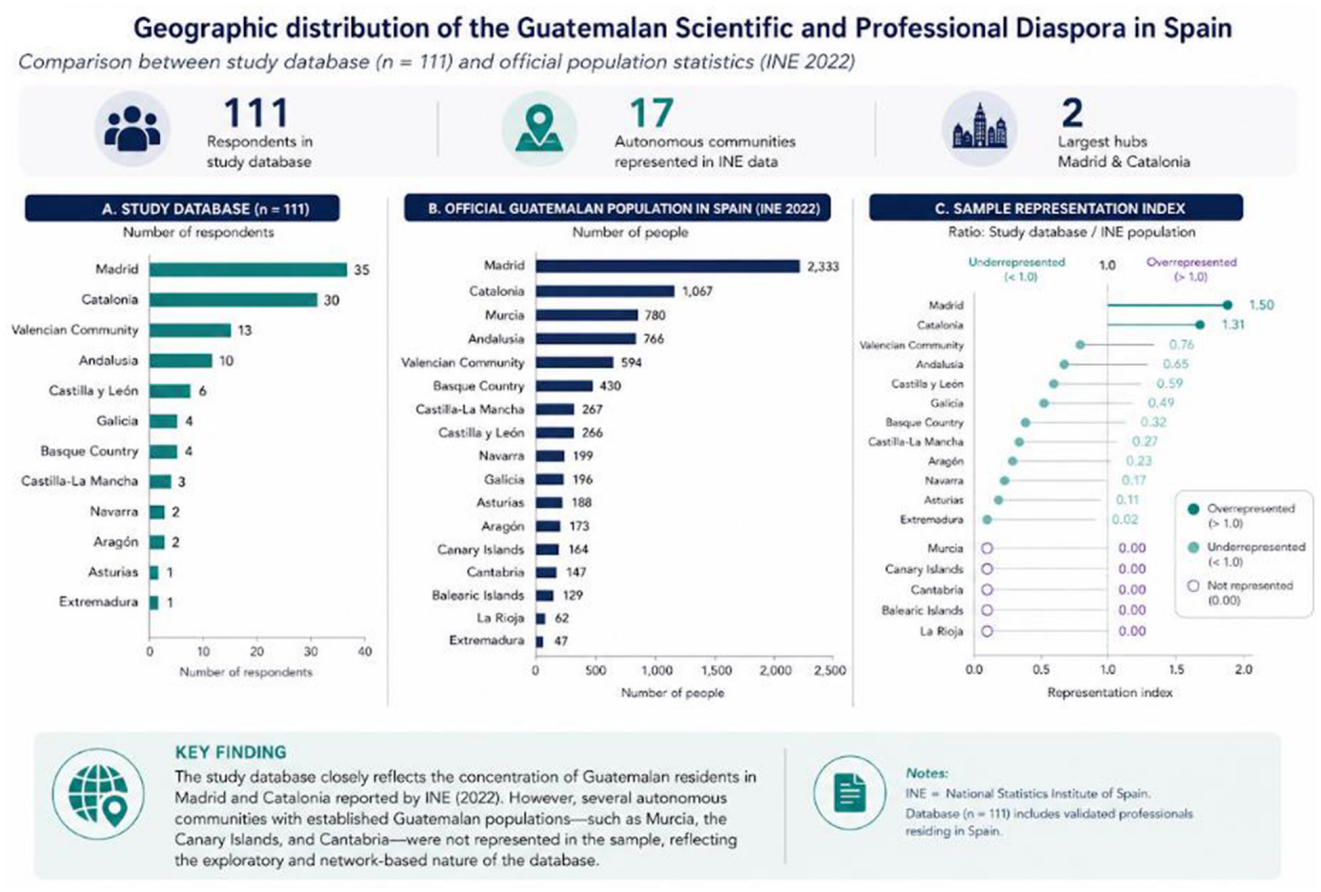
Geographic distribution of the Guatemalan scientific and professional Diaspora in Spain. Comparison between study database (*n* = 111) and official population statistics (INE 2022).

A total of 7,810 Guatemalans resided in Spain in 2022 (Instituto Nacional de Estadística, 2022). In both the INE report and this study, the majority of Guatemalans (*n* = 65, 44% of total population) of all professional and academic occupations primarily reside in Madrid and Catalonia (i.e., Barcelona). The next largest group (*n* = 23, 14%) of Guatemalan citizens reside in Andalucia and Valencia (Figure 2).

The concentration in urban centers fosters dense professional networks that are essential for consolidating transnational scientific communities (Meyer, 2011). Newly arrived GSPD members benefit from established peers within these networks who provide important support in overcoming structural, legal, cultural, and psychosocial challenges. This distribution aligns with settlement patterns observed among highly skilled migrants in other professional diasporas (OECD, 2024).

**Figure 3:**
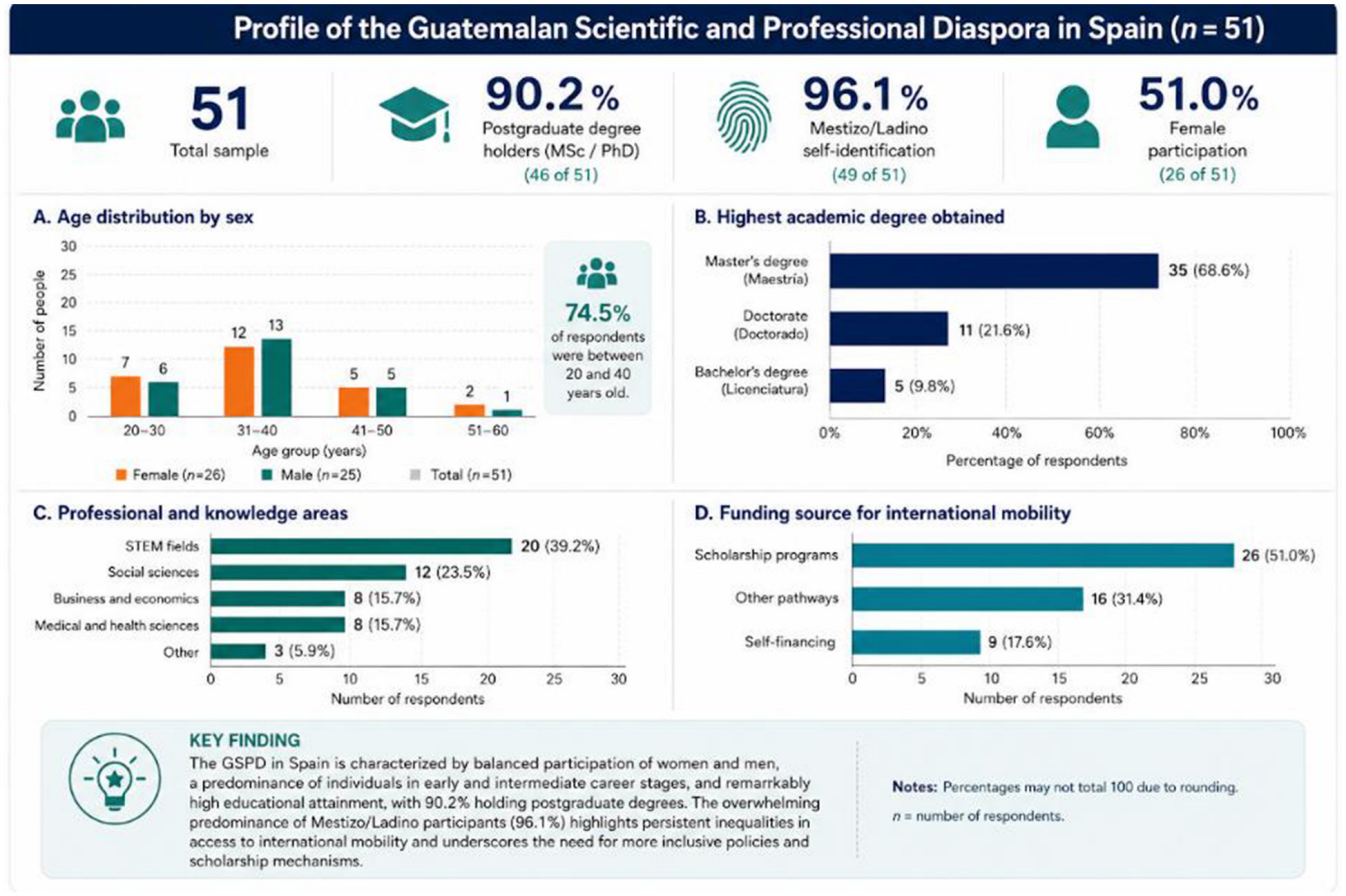
Profile of the Guatemalan scientific and professional Diaspora in Spain (*n* = 51).

The online questionnaire (*n* = 51) (Figure 3) shows a balanced gender distribution between women and men (*n* = 26 and *n* = 25) with no other gender identities reported. Most participants were young adults (20–30 years, *n* = 13, 26%) and adults (31–40, *n* = 23, 0 45%); 41–50, *n* = 11, 22%), with even distribution between genders. This migratory phenomenon has been observed in the broader migration of Central Americans to Western Europe, including Spain (Valenzuela Mejia, 2024).

Most participants indicated that they had migrated to improve their academic and professional skills. This suggests that they consider themselves to be at an early stage of their careers. Although participants were not directly asked whether they had families or children, none of the responses suggested that this academic and professional phase was shaped by family responsibilities or a particular life stage. There was a striking lack of ethnic diversity among the participants. 95% of participants identified as *mestizo* (*ladino*), a category Guatemalans use to identify the non-indigenous population.

#### 4.2.2 Educational attainment and career stage

**Figure 4:**
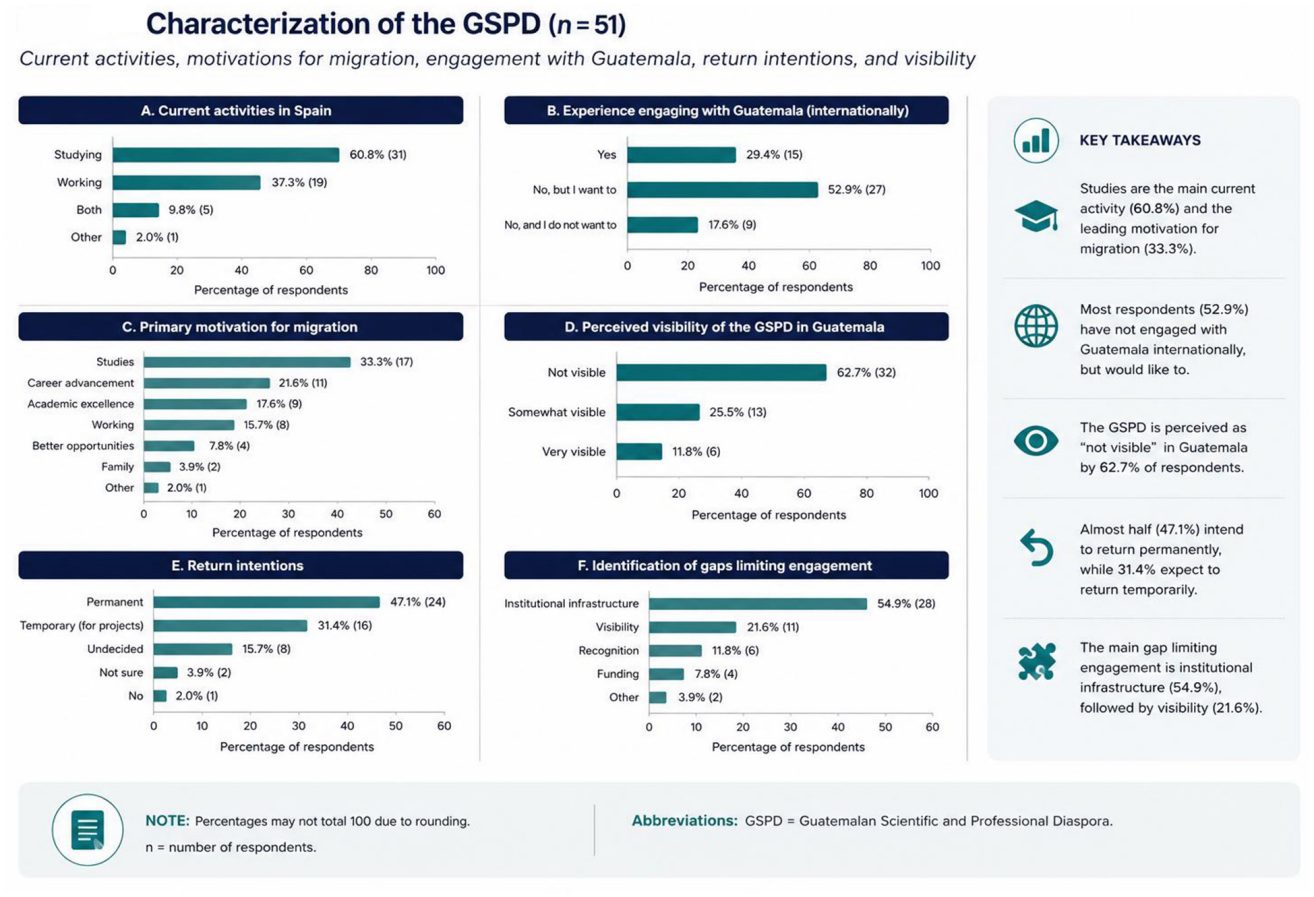
Characterization of the GSPD (*n* = 51). Current activities, motivations for migration, engagement with Guatemala, return intentions, and visibility.

Regardless of gender, members of the GSPD evaluated in this study are highly educated; most hold a master's degree, followed by a doctoral degree, and then bachelor's degree (Figure 4). This pattern aligns with global trends in academic and professional mobility, where the international migration of talent is closely linked to university networks, scholarship programs, and opportunities for advanced specialization (OECD, 2024; Valenzuela Mejia, 2024). Funding and mobility financing within members of the GSPD varied between genders and academic fields. In the case of women, mobility was through scholarship programs (*n* = 16), while men were more likely to self-finance or take a loan scholarship program (*n* = 14) mostly offered by Guatefuturo. STEM was the primary field of academic and professional development amongst both women and men, followed by social science fields, business, and medicine. Within STEM, slight differences were observed between women and men and across age groups. While men reported much higher occupations in engineering, physics, and computer sciences (*n* = 7) and biological and earth sciences (*n* = 3) than women (*n* = 5, *n* = 4, respectively), women reported higher occupation in medical sciences (*n* = 6) than men (*n* = 0). Social sciences showed no gender difference (each n=6). Interestingly, twice as many men (*n* = 6) than women, all within a self-financed scheme, were in business.

#### 4.2.3 Motivations for international mobility, professional integration and future perspectives

Academic mobility is the primary driver of migration to Spain. Half of the participants (*n* = 25) reported their reasons for migration were to advance in academic or professional careers and to achieve academic excellence (each *n* = 18). In contrast, less than a quarter (*n* = 12) reported their motivation was to improve livelihoods. Slightly more than half of the participants (n=26) reported to be studying, one third reported to be working (*n* = 15), while the rest did both (*n* = 9). This confirms the relevance of international educational mobility as a gateway to highly skilled migration. In many cases, these academic paths subsequently lead to permanent employment in the host country. Most participants do not foresee an immediate return to Guatemala. However, the literature on highly skilled migration suggests that this does not necessarily imply a permanent loss of human capital for the countries of origin. Rather, there is a shift from “brain drain” toward “knowledge circulation” (Meyer, 2011).

#### 4.2.4 Engagement with Guatemala

Participants showed strong interest in maintaining ties with Guatemala despite living abroad. The most frequently reported forms of engagement were mentorship, participation in diaspora networks, academic collaboration, and involvement in professional or development-oriented initiatives. These facilitate the transfer of knowledge, technology, and social capital between countries (UNESCO, 2006). The perception of social visibility of the GSPD is significant. The vast majority of respondents reported that the potential contribution of the professional diaspora remains invisible, particularly in the face of the predominance of public discourse focused on economic remittances.

#### 4.2.5 Institutional infrastructure and identified gaps limiting engagement

One of the most consistent findings of the survey was the weakness of the institutional linkage with the Guatemalan state. Participants described their relationship with the Guatemalan Diplomatic Mission (Embassy and consulates) as “minimal,” “nonexistent,” and “limited to administrative procedures.” Similarly, 89% of participants believe that there are no systematic records or mapping of the GSPD. This demonstrates a lack of institutionalization and reinforces their invisibility to the Guatemalan state.

Participants said the main obstacles to strengthening their links and enabling further engagement with Guatemala are: (i) lack of institutional platforms for coordination; (ii) scarce information on collaboration opportunities; (iii) absence of public policies geared toward the diaspora; (iv) limited funding for collaborative projects; and (v) weak structured professional networks. These results coincide with international studies that show that the impact of scientific diasporas depends largely on the existence of institutional liaison structures such as science diplomacy programs, transnational professional networks, and collaboration platforms (International Organization for Migration, 2019). Participants identified the following institutions that should be responsible for identifying, mapping, characterizing, and connecting the GSPD with Guatemala: MINEX, SENACYT, SEGEPLAN, and National Institute of Statistics (INE).

## 5 Discussion

Guatemala has a highly skilled pool of human capital in Spain with a strong potential to contribute to national development through transnational collaboration. However, to activate this potential, three institutional dimensions must be strengthened: (i) registration and mapping systems; (ii) transnational scientific and professional collaboration programs; and (iii) scientific diplomacy mechanisms and knowledge networks.

Focus group participants agreed that the diaspora is a heterogeneous community of professionals and scientists who share their Guatemalan origin and residence outside the country, but who maintain varying degrees of connection with Guatemala. They express this connection both in emotional and cultural terms and in the possibility of contributing to the country's scientific, academic, or professional development.

Discussions also revealed that migration to Spain has been strongly linked to opportunities for advanced training and professional specialization. This decision to migrate is initially associated with scholarship programs, postgraduate studies, or opportunities to join international academic networks. However, once these training processes are completed, job opportunities, institutional conditions for research, economic stability, and quality of life influence their decision to remain in the destination country. This suggests that most of the GSPD is in stages of professional consolidation. This coincides with international talent mobility patterns identified in the literature where highly qualified migration processes tend to occur during the early stages of professional development (Meyer, 2011).

The range of scholarship and support programs indicates a long-standing infrastructure for academic mobility and exchange between Guatemala and Spain. These connections are relevant and strategically necessary. This diaspora constitutes a reservoir of specialized knowledge, international research experience, and transnational networks that can be leveraged to strengthen national research systems, higher education, and innovation capacities. By systematically mapping and articulating the GSPD, it becomes possible to identify individuals and communities whose expertise can be mobilized for collaborative research, joint supervision, curriculum development, training programs, and evidence-based policy advice, both during their residence abroad and upon return to Guatemala.

The near absence of indigenous participants in the online survey reflects both the structural inequalities that continue to shape access and participation in Guatemala and limitations of the study design. Even when purposeful efforts were devoted to include voices from indigenous Guatemalan intellectuals in the diaspora, their visibility within the diaspora remains elusive. Indigenous people remain disproportionately affected by poverty (75% poverty level compared with 36% among non-Indigenous people) and social exclusion (ICEFI, 2017). In addition, the Guatemalan state allocates substantially fewer resources per indigenous person, spending USD 0.4 per day compared with USD 0.9 per day for each non-indigenous person (IACHR Inter-American Commission on Human Rights, 2025). These inequalities, combined with barriers related to geographic isolation, digital access, and institutional reach, mean that fewer indigenous people have the educational and economic opportunities to join the GSPD or even participate in an online-based survey. More broadly, this finding shows the lack of adequate education, academic, and development infrastructure to understand and respond to the needs of all territories, including indigenous ones. The mobility opportunities examined in this study are highly elitist and exclusionary, accessible mainly to only non-indigenous individuals. Therefore, it will require substantial support in order to close the persistent poverty and opportunity gap (Bonilla et al., 2025; McEwan and Trowbridge, 2007; Us et al., 2021). Despite their extended stays abroad, participants reported maintaining various ties with Guatemala: academic collaborations, mentoring students and young professionals, participation in networks of Guatemalans abroad, exchange of specialized knowledge, and involvement in projects or initiatives related to the country's development. However, these contributions tend to stem from individual initiatives or informal networks rather than from structured institutional mechanisms. Many participants pointed out the limited institutionalization in the relationship between the GSPD and Guatemalan state institutions. Participants noted the absence of formal platforms for identifying, mapping, and connecting Guatemalan professionals abroad and a lack of public policies aimed at leveraging the knowledge and international networks. There is a broad potential to strengthen transnational linkages through mentoring programs, structured professional networks, science diplomacy initiatives, and institutional mechanisms that facilitate knowledge transfer and international collaboration.

## 6 Conclusion

The responses in this study reinforce the literature on scientific diasporas that shows highly qualified migration does not necessarily imply an irreversible loss of human capital but rather can give rise to dynamics of knowledge circulation, brain linkage and connection. However, the study also reveals significant institutional limitations in harnessing the potential of the GSPD. These include a lack of formal mechanisms for collaboration between the diaspora and national institutions, the limited public visibility of this human capital, and the absence of specific policies aimed at facilitating transnational scientific and professional collaboration.

In this regard, strengthening strategies for engaging with the scientific diaspora–registration systems, professional networks, academic cooperation programs, and science diplomacy mechanisms–could significantly contribute to the development of the science, technology, and innovation ecosystem in Guatemala. Finally, given the exploratory nature of the study and the limited sample size, future research could broaden the analysis through comparative studies with other Guatemalan diasporas in different regions and the use of methodologies that allow for a deeper understanding of the specific mechanisms of knowledge transfer and international collaboration.

## 7 Limitations

The dataset analyzed in this study includes 51 complete responses. The participation was voluntary and the questionnaire was distributed through professional and academic networks, the sample is a non-probability, self-selection sample. Consequently, the results should be interpreted as an exploratory characterization of a highly qualified segment of the Guatemalan diaspora, and not as a statistical representation of the entire Guatemalan population residing in Spain.

## Data Availability

The raw data supporting the conclusions of this article will be made available by the authors, without undue reservation.
