# Supplementary material for: Guatemala scientific and professional diaspora in Spain: an initial characterization
Source: Front Res Metr Anal. 2026 Jul 16;11:1860284. doi: 10.3389/frma.2026.1860284 (PMC13422382; doi:10.3389/frma.2026.1860284)
Supplement: Supplementary file 1 [file Table_1.docx]

# **Questionnaire for the Guatemalan Scientific and Professional Diaspora (DCPG) in Spain**

**Consultation period: January 15 – March 15, 2026**

We invite: i) Guatemalans, ii) Guatemalans residing abroad, iii) those engaged in professional, academic, or research activities, and iv) those with current or potential collaborations in Guatemala, to complete this consultation questionnaire.

This consultation stems from the project ‘Connecting the Guatemalan Scientific and Professional Diaspora (DCPG)’, led by the National Secretariat of Science and Technology in Guatemala (SENACYT) and the Academy of Medical, Physical, and Natural Sciences. The initiative seeks to strengthen institutional capacities to map, connect, and collaborate strategically with the Guatemalan Scientific and Professional Diaspora (DCPG), promoting its contribution to national development in science, technology, and innovation. The project is supported by two cooperating entities. On one hand, the Capacity Development Laboratory (CDL) of the EU Global Diaspora Programme (EUDiF), a program implemented by the International Centre for Migration Policy Development (ICMPD) and funded by the European Union. On the other hand, the Spanish Cultural Center in Guatemala (CCEG). Researchers Kleinsy Bonilla and Claudia Romero-Oliva are coordinating the process. For inquiries, comments, or suggestions, please write to diasporaguatemala@senacyt.gob.gt.

Your collaboration will be invaluable in developing an analysis of the characteristics of the Guatemalan scientific and professional community residing abroad. We respectfully request your support by completing this survey, and we guarantee the confidentiality of your personal data. Your participation will expand our knowledge of the Guatemalan Scientific and Professional Community (DCPG) and generate input that contributes to understanding their trajectories and ties to their country of origin. This information contributes to strengthening the analysis and visibility of the diaspora, which is important for placing this issue on the public agenda—a key aspect for instruments like this to be effectively integrated into decision-making processes.

By completing the form, you authorize the research team to store and process your data for the purposes described above, in aggregated, anonymized form, without any traceability of personal identities. Completing the questionnaire takes 20 minutes.

# Section I - GENERAL INFORMATION - Identification

General information on members of the DCPG (Guatemalan scientific and professional diaspora)

Email address you check most often:

Full name:

Age range:

Younger than 20

20 -30

31-40

41-50

51-60

Older than 60

Gender:

Female

Male

I prefer no to say

Other

Digital Profiles

LinkedIn

GoogleScholar / other professional digital profile

Social Networks

Name of the institution where you obtained your last academic degree

Country of the institution where you obtained your last academic degree

Highest academic degree obtained - What is your highest or most recent academic degree?

Doctorate (PhD)

Master's Degree

Postgraduate Diploma

Bachelor's Degree

Other

Ethnic identity

Mestizo / Ladino

Indigenous (Maya)

Xinca

Garifuna

I prefer not to respond

Other

Professional, scientific, and technological field in which you currently work

Agricultural sciences

Astronomy

Earth and space sciences

Biological systems and organisms

Chemical sciences

Computer science and information technology

Engineering sciences

Mathematics

Medical and health sciences (including neuroscience)

Physical sciences

Structural, cellular, and molecular biology

Social sciences

Business, commercial sector

Other:

Which of the following best describes your main activity at this time?

Studying

Working full-time

Combining study and work

Retired

Other

Career or professional stage:

Early stage: up to 5 years working in your area of ​​expertise

Mid-stage: 6 to 10 years working in your area of ​​expertise

Established stage: 11 to 15 years working in your area of ​​expertise

Senior stage: 16 years and older working in your area of ​​expertise

Region of your current residency

North America (Canada, USA, Mexico)

Europe

Latin America and the Caribbean

Oceania (Australia, New Zealand)

Asia

Other

Country of current residency

City of Current Residency

# Section II - Characterization - Mapping

Reasons for migration to your country of residence: select those that apply

Higher education studies with a scholarship

Self-funded higher education studies

Work-related motivations to pursue paid employment in Spain

Personal reasons, family reunification

Other reasons (please specify)

If you have completed university studies in your country of residence, what was or is your main source of funding?

Scholarship awarded by the Guatemalan government

Guatefuturo

Scholarship awarded by an international cooperation program

Scholarship from a university in your country of residence

I have not studied in my host country

Other

Scholarship program(s) that supported your studies. Please indicate the scholarship programs that have supported your education (e.g., Erasmus Scholarships, Carolina Foundation, Marie Curie Program, Chevening, KOICA, etc.). Please list all that apply.

Prospects for remaining abroad/returning to Guatemala

My plan is to continue residing outside of Guatemala indefinitely

My plan is to continue residing outside of Guatemala temporarily

I plan to return to Guatemala in the short term (within the next year

I plan to return to Guatemala in the medium term (within the next two years)

Other:

To your knowledge, are there any records, databases, mappings, or characterizations of the DCPG (Guatemalan migrants with high levels of education, developing professional, scientific, or research activities)?

Yes

No

If your answer to the previous question is yes, please share details. What type of registration, who is managing it, and access links

In your opinion, which institution/entity should be responsible for generating DCPG data and indicators?

The Ministry of Foreign Affairs MINEX

The National Secretariat for Science and Technology SENACYT

The National Secretariat for Planning and Programming of the Presidency SEGEPLAN

The San Carlos of Guatemala University

The National Institute of Statistics INE

Other:

# Section III - Conexion with Guatemala

Main document I use to identity myself to others: select only one

Guatemalan passport

Foreign passport

Guatemalan National Identity Document (DPI)

Identity document issued by another country

Other

Tax declaration: (This question seeks to determine if you maintain this type of connection with Guatemala; the answer is optional)

I file tax returns exclusively in my host country

I file tax returns exclusively in Guatemala

I file tax returns in both Spain and Guatemala

Other

Tax declaration: (This question seeks to determine if you maintain this type of connection with Guatemala; the answer is optional)

My Tax Identification Number (NIT or RTU) is active with the Guatemalan Tax Administration (SAT)

My Tax Identification Number (NIT or RTU) is inactive with the Guatemalan Tax Administration (SAT)

Other

Professional practice and legal requirement of professional registration in Guatemala:

I joined a professional association in Guatemala but I'm not active.

I joined a professional association in Guatemala and I'm active.

I never joined a professional association in Guatemala.

Finance and banking connexion with Guatemala

I have one or more active bank accounts in Guatemala, of which I am the account holder.

I have one or more bank accounts in Guatemala, but I don't use them.

I don't have any bank accounts in Guatemala.

How would you categorize your relationship (service/attention provided to you) with the Guatemalan Diplomatic Mission and consular services in your country of residence?

-None, I have no relationship

-Minimal, only what is strictly necessary: ​​passport renewal, DPI (Guatemalan national identification card), consular services

-Basic, in addition to consular services, I have received support with document processing, legal documents/apostilles

-Active, I receive regular information from the Embassy, ​​invitations to participate in activities with the Guatemalan community in Spain

-Other

Other connections with Guatemala (Guatemalan Social Security Institute IGSS / Pension scheme):

I continue to make contributions to the IGSS.

I started making contributions to the IGSS, but my contributions have been suspended since I moved overseas

I maintain contributions to private pension plans.

I never registered with the Guatemalan Social Security Institute (IGSS).

Connecting with other Guatemalans in your country of residence:

-I don't know any other Guatemalans living in my host country

-I participate in informal networking spaces, WhatsApp groups, Facebook groups, and other self-organized social media.

-I participate in structured and systematic activities with part of the Guatemalan community living in my host country

-If you have any other options, please provide details.

# Section IV - Engagement with Guatemala

What forms of engagement have you had or do you have with Guatemala in your professional area or field of knowledge? Select all that apply.

-Collaboration on research projects with Guatemalan institutions

-Mentoring and talent development in Guatemala

-Participation in networks of Guatemalan scientists and/or professionals abroad

-Consulting for public institutions in Guatemala

-Collaborations with private sector entities

-I am currently unengaged, but would like to engage

-I am currently unengaged and have no interest in establishing one

-Other (please specify)

Which do you consider to be the main obstacles to strengthening your ties with Guatemala?

-Lack of information on collaboration opportunities

-Lack of organizations or institutions that facilitate connections with the scientific and/or professional diaspora

-Limitations in funding for joint projects

-Lack of public policies that facilitate connections with the scientific and/or professional diaspora

-Scarcity of formal networks or platforms to connect with scientists and/or professionals abroad

-Other (please specify)

Perception in Guatemala regarding the visibility of the Guatemalan Scientific and Professional Diaspora (DCPG). In their opinion, Guatemalan society (institutions, media) recognizes the existence and potential contribution of the Guatemalan scientific and professional diaspora (beyond remittances).

Yes

No

Please elaborate on your answer to the previous question (DCPG visibility)

What kind of initiatives would motivate you to strengthen your ties with Guatemala?

Creation of specific funds for collaborative projects between the diaspora and the home country

Mobility programs that facilitate short research stays, professional development courses, and teaching

-Digital platforms to connect Guatemalan scientists and/or professionals abroad with national institutions

-Spaces for scientific and professional diplomacy that contribute to the development of public policies

-Improved collaboration with consulates/embassies of Guatemala that have information and outreach programs

-Other (please specify)

Have you participated in any institutional initiatives to link the diaspora with Guatemala?

Yes, I currently participate actively.

Yes, but sporadically.

No, but I would like to.

No, and I have no interest in participating.

If you have any professional ties to Guatemala, through what type of institution have you managed your ties?

-Guatemalan universities or research centers

-Government institutions (ministries, secretariats, embassies, etc.)

-Non-governmental organizations (NGOs) or scientific and professional foundations

-Networks or associations of the Guatemalan scientific and professional diaspora

- I have not arranged my engagement through any institution or organization

- Other (please specify)

How effective has the mediation of these institutions been?

-Very effective: It significantly facilitated my participation and collaboration.

-Moderately effective: I had support, but with some limitations.

-Somewhat effective: There were barriers that hindered the connection.

-Not at all effective: I received no support or the connection did not materialize.

-I have no experience connecting with mediation from institutions/organizations.

What actions do you think could improve the mediation of institutions/organizations to strengthen the link between the scientific diaspora and Guatemala?

Specific engagement programs

Greater dissemination of opportunities

Reduction of bureaucratic barriers

Increased financing

Other:

What do you consider to be the main barriers that hinder the engagement with Guatemala?

-Lack of Information

-Shortage of funding

-Lack of networking / platforms for interaction

-Administrative/bureaucratic barriers

- Lack of interest/recognition of this type of link in Guatemala

- Other

Have you tried to connect with institutions in Guatemala and encountered difficulties?

Yes, I tried to get involved, but I encountered many barriers that prevented it.

Yes, but I only encountered some difficulties that I was able to overcome.

I haven't tried, but I would like to.

I haven't tried and I have no interest in getting involved.

What measures would reduce the barriers to engagement with Guatemala?

-Clear and accessible mechanisms

-Further support from the Government

-Financing programs

-Strengthening networks and partnerships

-Other

Are you in contact with a university or academic institutions in Guatemala?

Yes, I maintain an active relationship with my university and/or academic and/or professional institutions in Guatemala.

Yes, but sporadically or indirectly.

No, but I would like to establish contact.

No, and I have no interest in becoming involved.

Through what channels would you be willing to share information or collaborate?

-Email or newsletters

-Digital groups or platforms (WhatsApp, LinkedIn, Telegram, specialized forums)

-In-person or virtual academic and professional events

-Professional and scientific social networks

-I am not interested in sharing information or collaborating
